# Effect of Cognitive Training in Fully Immersive Virtual Reality on Visuospatial Function and Frontal-Occipital Functional Connectivity in Predementia: Randomized Controlled Trial

**DOI:** 10.2196/24526

**Published:** 2021-05-06

**Authors:** Jae Myeong Kang, Nambeom Kim, Sook Young Lee, Soo Kyun Woo, Geumjin Park, Byeong Kil Yeon, Jung Woon Park, Jung-Hae Youn, Seung-Ho Ryu, Jun-Young Lee, Seong-Jin Cho

**Affiliations:** 1 Department of Psychiatry Gil Medical Center Gachon University College of Medicine Incheon Republic of Korea; 2 Brain Health Center Gil Medical Center Incheon Republic of Korea; 3 Biomedical Engineering Research Center Gachon University Incheon Republic of Korea; 4 Department of Psychiatry Gyeonggi Provincial Medical Center Suwon Hospital Suwon Republic of Korea; 5 Department of Game Engineering and IT Convergence Engineering Graduate School of Gachon University Seongnam Republic of Korea; 6 Department of Counseling Psychology Cha University Seongnam Republic of Korea; 7 Department of Psychiatry, School of Medicine Konkuk University Medical Center Konkuk University Seoul Republic of Korea; 8 Department of Psychiatry SMG-SNU Boramae Medical Center Seoul National University College of Medicine Seoul Republic of Korea

**Keywords:** virtual reality, cognitive training, visuospatial function, fMRI, visual network, mild cognitive impairment

## Abstract

**Background:**

Cognitive training can potentially prevent cognitive decline. However, the results of recent studies using semi-immersive virtual reality (VR)-assisted cognitive training are inconsistent.

**Objective:**

We aimed to examine the hypothesis that cognitive training using fully immersive VR, which may facilitate visuospatial processes, could improve visuospatial functioning, comprehensive neuropsychological functioning, psychiatric symptoms, and functional connectivity in the visual brain network in predementia.

**Methods:**

Participants over 60 years old with subjective cognitive decline or mild cognitive impairment from a memory clinic were randomly allocated to the VR (n=23) or the control (n=18) group. The VR group participants received multidomain and neuropsychologist-assisted cognitive training in a fully immersive VR environment twice a week for 1 month. The control group participants did not undergo any additional intervention except for their usual therapy such as pharmacotherapy. Participants of both groups were evaluated for cognitive function using face-to-face comprehensive neuropsychological tests, including the Rey-Osterrieth Complex Figure Test (RCFT) copy task; for psychiatric symptoms such as depression, apathy, affect, and quality of life; as well as resting-state functional magnetic resonance imaging (rsfMRI) at baseline and after training. Repeated-measures analysis of variance was used to compare the effect of cognitive training between groups. Seed-to-voxel–based analyses were used to identify the cognitive improvement–related functional connectivity in the visual network of the brain.

**Results:**

After VR cognitive training, significant improvement was found in the total score (F_1,39_=14.69, *P*=.001) and basic components score of the RCFT copy task (F_1,39_=9.27, *P*=.005) compared with those of the control group. The VR group also showed improvements, albeit not significant, in naming ability (F_1,39_=3.55, *P*=.07), verbal memory delayed recall (F_1,39_=3.03, *P*=.09), and phonemic fluency (F_1,39_=3.08, *P*=.09). Improvements in psychiatric symptoms such as apathy (F_1,39_=7.02, *P*=.01), affect (F_1,39_=14.40, *P*=.001 for positive affect; F_1,39_=4.23, *P*=.047 for negative affect), and quality of life (F_1,39_=4.49, *P*=.04) were found in the VR group compared to the control group. Improvement in the RCFT copy task was associated with a frontal-occipital functional connectivity increase revealed by rsfMRI in the VR group compared to the control group.

**Conclusions:**

Fully immersive VR cognitive training had positive effects on the visuospatial function, apathy, affect, quality of life, and increased frontal-occipital functional connectivity in older people in a predementia state. Future trials using VR cognitive training with larger sample sizes and more sophisticated designs over a longer duration may reveal greater improvements in cognition, psychiatric symptoms, and brain functional connectivity.

**Trial Registration:**

Clinical Research Information Service KCT0005243; https://tinyurl.com/2a4kfasa

## Introduction

Dementia is a major neurodegenerative disorder, affecting approximately 10% of older people [1]. Cognitive, psychological, and behavioral deterioration are typical manifestations of dementia, ultimately resulting in functional impairments and disability [2]. The individual and societal burden of dementia is accelerating rapidly compared to other diseases [1,3]; however, due to the unclear mechanisms and multifactorial pathology underlying the development and progression of dementia, only symptomatic treatments are currently available [4].

To date, many researchers have suggested that prevention is crucial, and have identified risk and protective factors associated with dementia, as well as preventive strategies [5]. According to a recent large study, one-third of Alzheimer disease (AD) cases are attributable to potentially modifiable risk factors such as educational attainment, vascular factors, and depression [6]. Additionally, lifelong exposure to cognitively and mentally engaging activities has been shown to protect against cognitive decline [7], and performance of cognitively stimulating activities in advanced age was associated with better cognitive function [8]. Accordingly, recent cognitive training studies have shown that repeated practice of exercises to restore brain and cognitive reserves resulted in small to moderate positive improvements in cognition in patients with mild to moderate dementia [9].

Advances in computer sciences and information and communication technology (ICT) have resulted in increased availability and accessibility of computerized cognitive training. Although conclusive results have yet to be found, preliminary studies have reported improvements in trained and nontrained cognition, and enhanced brain activity in related regions after computerized cognitive training in individuals with mild cognitive impairment (MCI) [10-12]. Working memory training was effective in improving verbal memory and hippocampal activation in patients with MCI [11], and exposure to a driving video game resulted in increased ability to control the vehicle that was related to midline frontal theta power in older people [12]. Moreover, emerging ICT applications using virtual reality (VR) have resulted in evolutions in health care, including cognitive and behavioral therapy [13]. VR can offer interventions in flexible and real world–like environments, facilitating visuospatial function through learning and transference outcomes [14], highlighting a role for cognitive training in a virtual environment in basic research and clinical practice. Owing to the lack of knowledge and dearth of experiments on VR-based cognitive training, especially the fully immersive type [15,16], further studies are needed to ascertain its potential therapeutic efficacy.

Recently, the number of neuroimaging studies attempting to reveal the underlying neural mechanisms associated with cognitive decline has increased [17,18]. Functional connectivity studies using resting-state functional magnetic resonance imaging (rsfMRI) have identified networks temporally coinciding with spatially distant neurophysiological events that are intrinsically coherent during a resting state such as the default-mode network [19]. We considered that functional connectivity studies using rsfMRI may be able to reveal the neural mechanism, especially in the visual network, responsible for the observed cognitive improvements following VR cognitive training, as such training is based on the cognitive reserve hypothesis associated with functional neural networks [20].

To test this hypothesis, we performed a preliminary randomized controlled trial to determine the efficacy and mechanisms of VR cognitive training in a predementia state. We aimed to ascertain the effects of VR multidomain cognitive training on visuospatial function, comprehensive neuropsychological function, and psychiatric symptoms in predementia. Moreover, we examined the hypothesis that cognitive improvement could be related to increased functional connectivity in the visual network of the brain.

## Methods

### Participants

Participants over 60 years old in a predementia state (ranging from subjective cognitive decline to MCI) were prospectively recruited between May and December 2019 from the memory clinic of Gachon University Gil Medical Center, Republic of Korea. Among 58 individuals who were assessed for eligibility using structured clinical interviews and brain MRI, four participants were excluded due to cerebral infarction on MRI (n=2), severe white matter hyperintensity on MRI (n=1), and history of a recent dental implant surgery (n=1). Nine participants voluntarily withdrew from the study due to an acute medical condition (n=2), hospitalization of a family member (n=1), scheduling conflict (n=1), and unknown personal reasons (n=5). Finally, a total of 45 participants were randomly assigned to either the VR group or the control group.

All participants had subjective cognitive complaints, including memory decline, but did not meet the criteria for diagnosis of a major neurocognitive disorder based on the Diagnostic and Statistical Manual of Mental Disorders (5th edition) [2]. Participants were classified as having subjective cognitive decline according to the corresponding research criteria and five cognitive domain scores such as attention, language, visuospatial, memory, and frontal executive functions above –1.5 SD [21,22]. Participants were classified as having MCI according to the Petersen criteria [23]. Screening evaluation of the participants was performed by a board-certified psychiatrist (JK) and a clinical neuropsychologist (SL).

The exclusion criteria for the participants were as follows: (i) Korean version of Mini-Mental State Examination (MMSE) score <20; (ii) impaired activities of daily living; (iii) comorbidity of severe medical or surgical conditions; (iv) major psychiatric disorders; (v) history of any kind of dementia; (vi) history of neurodegenerative disorders, including Creutzfeldt-Jakob disease, Pick disease, Huntington disease, Parkinson disease, inflammation associated with HIV, and syphilis; (vii) structural abnormalities on MRI such as intracranial hemorrhage, cerebral, cerebellar, or brainstem infarction, hydrocephalus, traumatic brain injury, severe white matter hyperintensity, tumors, multiple sclerosis, or vasculitis; and (viii) inability to use the VR system.

Information on study objectives, group allocation, cognitive intervention, brief study protocol, risks and benefits, and confidentiality was given to all participants before enrollment. All participants provided offline written informed consent, and the Institutional Review Board of Gachon University Gil Medical Center approved this study (GCIRB2018-396).

### Study Design

This was an open-label, randomized controlled trial (KCT0005243) that aimed to investigate the efficacy of a fully immersive VR cognitive training program on visuospatial function in older people with risk for dementia (Multimedia Appendix 1). Participants were randomly assigned to either the VR or the control group. The unblinded randomization was performed by drawing lots with the participants present. Participants in both groups were evaluated for visuospatial function, comprehensive neuropsychological function, and psychiatric symptoms, and underwent rsfMRI before and after the 1-month cognitive training. The participants in the VR group underwent VR cognitive training twice a week for a total of eight sessions in addition to their usual therapy such as pharmacotherapy for the prevention of dementia (eg, choline alfoscerate and cholinesterase inhibitor); the participants in the control group did not undergo any additional intervention except for their usual therapy such as pharmacotherapy.

### VR Cognitive Training

The multidomain VR cognitive training program was developed between November 2018 and April 2019 by the authors who are board-certified geriatric neuropsychiatrists and clinical neuropsychologists with expertise. The VR cognitive training program consisted of multiple games involving multidomain cognitive tasks to assess: (i) attention (to find differences), (ii) executive function and memory (to select items needed to perform certain tasks), (iii) working memory and ability to perform mathematical calculations (to prepare an exact amount of money), (iv) visuospatial orientation (to find a path using a memorized map), (v) visuospatial function (to spatially place furniture exactly based on a memorized drawing), (vi) verbal memory (to remember certain words), (vii) visual memory (to remember specific flags and symbols), and (viii) processing speed and working memory (to catch animals in a certain order). All virtual environments were fully immersive 3D settings allowing for feelings of increased presence and visuospatial stimulation; training was accompanied by game elements to increase the interest and motivation of the participants. Representative images of the VR training program are presented in Multimedia Appendix 2.

Each session lasted approximately 20-30 minutes. The VR training took place using a head-mounted Oculus Rift CV1 display, with Oculus Touch controllers held in both of the participant’s hands. Each training session was performed with the participant in a seated position, and the difficulty level increased throughout the study period from easy to difficult (levels 1-4), with two sessions at each difficulty level. All procedures were performed in the memory clinic of Gachon University Gil Medical Center and were guided by a certified clinical neuropsychologist (SL) in addition to automatic verbal and visual messages from the program. There were no revisions, updates, or breaches of the program during the study period. This program was used exclusively in this study and is not available for commercial use.

### Procedures and Outcome Measures

All participants underwent face-to-face comprehensive neuropsychological tests and evaluations using psychiatric scales, as well as rsfMRI at baseline and after the VR cognitive training period. Baseline evaluations of diagnostic criteria included global and functional scales such as the Korean version of the MMSE, Clinical Dementia Rating (CDR), CDR Sum of Boxes (CDR-SOB), global deterioration scale, and instrumental activities of daily living scales.

The primary outcome was the effect of the VR cognitive training on visuospatial function measured by the Rey-Osterrieth Complex Figure Test (RCFT) copy task, which has been validated in the Korean population [24,25]. Basic components, including a large rectangle (1 point), diagonal cross (1 point), horizontal midline of a large rectangle (1 point), and vertical midline of a large rectangle (1 point), were also evaluated because they are considered important in qualitative aspects [26,27]. The neuropsychologist (GP) who scored the RCFT copy task was blinded to the randomization.

The secondary outcomes concerned the effect on comprehensive neuropsychological function; psychiatric symptoms such as affect, apathy, quality of life (QoL), and depression; and functional connectivity in the visual network of the brain.

The neuropsychological tests consisted of the MMSE and subtests from the comprehensive neuropsychological test battery [25]. Attention was assessed by the digit span forward and backward test and Trail Making Test (TMT) part A [25]. The Korean version of the Boston Naming Test (K-BNT) was used to assess language ability [25,28]. Memory was assessed by measuring performance on three tasks of the Seoul Verbal Learning Test (SVLT): immediate recall, delayed recall after 20 minutes, and recognition [25]. Frontal executive function was assessed by phonemic word fluency testing, the TMT-B, and the Stroop Color Test [25]. All neuropsychological test results were adjusted for age and years of education, and are presented as standardized *z*-scores.

Noncognitive psychiatric symptoms that typically start to decline in the early dementia stage were also assessed [29]. Depressive symptoms were evaluated by the validated 30-item Geriatric Depression Scale (GDS), including questions pertaining to mood, anxiety, energy, satisfaction, hopefulness, inattention, and sleep quality [30,31]. The GDS comprises a series of binary yes/no questions (scored as 1 or 0, respectively), with higher scores indicating severe depression. Apathy was evaluated by the validated 18-item Apathy Evaluation Scale (AES), including items pertaining to emotional affect, behavior, and cognitive apathy [32,33]. Items of the AES are rated on a 4-point Likert scale, with a low score indicating severe apathy. Affect was evaluated by the Positive and Negative Affect Schedule (PANAS), which consists of 10 items to assess positive affect (PANAS-P) measures such as alertness and enthusiasm and 10 items to assess negative affect (PANAS-N) such as lethargy and feelings of sadness [34,35]. Each of the PANAS items is rated from 1 (not at all) to 5 (very much), with higher scores indicating higher affect. Participants’ QoL was evaluated by the QoL-AD scale, which has been validated for use in people with dementia, including 13 subjective rating items to assess physical health, living situation, relationships with friends, and the ability to engage in leisure activities [36,37]. Items of the QoL-AD are assessed on a 4-point Likert scale, with higher scores indicating better QoL.

The Simulator Sickness Questionnaire (SSQ) was administered after each session to evaluate tolerability of the VR cognitive training program [38]. Simulator sickness refers to side effects from virtual environment usage, and is also called cybersickness [39,40] and VR sickness [41]. The SSQ consists of 16 items yielding three subscales (nausea, oculomotor, and disorientation) and a total severity score, with high scores indicating increased symptoms. The levels of interest and satisfaction were also assessed on a Likert scale ranging from 0 to 100 after the period of VR cognitive training in a face-to-face manner.

### MRI Acquisition

A 3-Tesla whole-body Siemens scanner (TrioTim syngo) was used for functional image acquisition with an interleaved T2*-weighted echo-planar imaging gradient echo sequence (repetition time/echo time=2500/25 milliseconds, flip angle=90°, slice thickness=3.5 mm, in-plane resolution=3.5×3.5 mm, matrix size=64×64) with a 12-channel birdcage head coil. For each participant, 160 functional volumes were acquired at the pretraining and posttraining time points. After rsfMRI, an anatomical image was acquired using a high T1-weighted 3D-gradient echo pulse sequence with magnetization-prepared rapid gradient echo (repetition time/echo time/inversion time=1900/3.3/900 milliseconds, flip angle=9°, slice thickness=1.0 mm, in-plane resolution=0.5×0.5 mm, matrix size=416×512). T1-weighted images were acquired only at the pretraining time point.

### Functional Connectivity Analyses With rsfMRI

Preprocessing of the rsfMRI data was performed using Statistical Parametric Mapping software version 12 (Wellcome Trust Centre for Neuroimaging). First, a slice-timing correction was applied and the center of each image was relocated near the anterior commissure. Second, rsfMRI and T1-weighted images were imported into CONN FC toolbox v19c [42] for further preprocessing. To correct for between-scan rigid body motion, the functional images were realigned to the first image in the time series. The functional images were coregistered with anatomical images and spatially normalized to the Montreal Neurological Institute space using a transformation matrix derived from the T1-weighted anatomical image segmentation. The functional images were then resliced to 2×2×2 mm and spatially smoothed using an 8-mm full width at half maximum Gaussian kernel.

All preprocessed rsfMRI images were bandpass-filtered (0.008-0.09 Hz), and physiological and other spurious noise sources in the blood oxygenation level–dependent signal were removed using the anatomical component-based noise correction strategy implemented in CONN [43]. Outliers were calculated using the Artifact Detection Tools toolbox [44], and six motion correction parameters obtained from realignment were also modeled as nuisance covariates. The seed-to-voxel analyses were performed in the visual network with four cortical seed regions (right visual lateral, left visual lateral, visual medial, and visual occipital cortices) with predefined regions of interest based on the Harvard-Oxford atlas (fMRIB Software Library) [45]. Seed-based analyses were adjusted for age, years of education, sex, CDR-SOB, depressive symptoms, and pharmacotherapy. The mean time series for each seed region was calculated and then correlated with the time courses of all other voxels in the brain for each participant.

### Sample Calculation and Statistical Analyses

Sample calculation was based on a recent meta-analysis on the effectiveness of VR for people with MCI or dementia that produced small-to-medium effect sizes using a random-effects model (effect size=0.29) from a total of 11 studies [15]. Assuming an attrition rate of 20%, a total sample size of 32 patients (16 per treatment group) would provide 0.8 power at a two-sided α error of .05. Power analysis was performed with G*Power software version 3.1.9.2.

Comparisons of demographic and clinical variables between the two groups were performed using independent *t* tests, the Mann-Whitney *U* test, or the χ^2^ test. The paired *t* test was used in within-group comparisons of pretraining and posttraining measures. Repeated-measures analyses of variance was used to find the group interaction of the VR cognitive training on neuropsychological function and psychiatric symptom scales after adjusting for age, years of education, sex, CDR-SOB, depressive symptoms, and pharmacotherapy. Age and years of education were not adjusted in analyses with comprehensive neuropsychological test results that are presented as age- and years of education–adjusted *z*-scores. All statistical analyses were performed with SPSS software version 23 (SPSS Inc), with a significance level assessed at *P*<.05 (two-tailed).

For rsfMRI data, Pearson correlation coefficients were converted to normally distributed scores using the Fisher *r*-to-*z* transformation. Group-level comparisons between the VR and control groups were performed using a general linear model in which improved cognitive task score was used as an explanatory variable and the posttraining minus pretraining *z*-transformation value was used as a dependent variable after adjusting for age, sex, years of education, CDR-SOB, depressive symptoms, and pharmacotherapy. The statistical thresholds for significance were set at voxel-wise uncorrected *P*<.001 and cluster-wise corrected *P*<.05 to correct for false-positive rates.

## Results

### Participants

**Figure 1:**
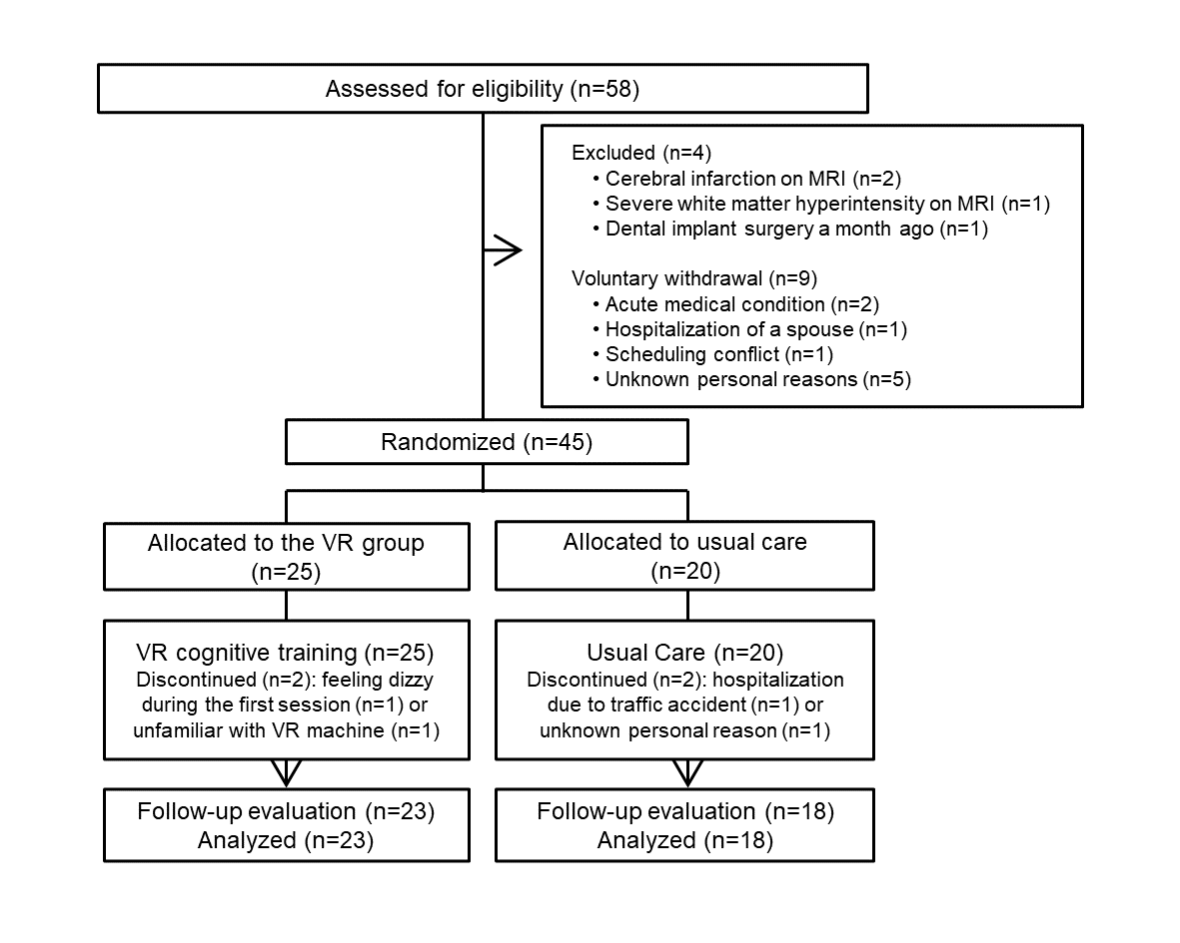
Trial flow chart. VR: virtual reality; MRI: magnetic resonance imaging.

Of the 45 participants who were randomly allocated to the VR (n=25) or the control (n=20) group, 41 participants completed the study. After allocation, two participants of the VR group dropped out of the study due to dizziness (n=1) and unfamiliarity with the VR machine during the first session (n=1). Two participants of the control group dropped out because of hospitalization due to a traffic accident (n=1) and unknown personal reasons (n=1). Ultimately, 41 participants were included in the analyses. The trial flow chart is presented in Figure 1.

### Demographic and Clinical Characteristics

Table 1 presents the detailed demographic and clinical characteristics of the study participants. Among the 41 participants, 23 (56%) and 18 (44%) were assigned to the VR and control groups, respectively. Participant age was around 75 years, and they were predominantly women. No group differences were found in the baseline diagnostic evaluation.

**Table 1:** Demographic and clinical characteristics of all study participants.

Characteristic	Total (N=41)	VR^a^ group (n=23)	Control group (n=18)	χ^2^ or *U*^b^	*P* value
Age (years), mean (SD)	74.51 (5.81)	75.48 (4.67)	73.28 (6.96)	–1.13	.26
Sex (female), n (%)	29 (70.7)	17 (73.9)	12 (66.7)	0.26	.61
Education (years), mean (SD)	8.07 (4.39)	7.70 (4.10)	8.56 (4.83)	–0.01	.99
MMSE^c^, mean (SD)	26.24 (2.85)	26.22 (2.91)	26.28 (2.87)	0.09	.93
CDR^d^, mean (SD)	0.41 (0.22)	0.41 (0.19)	0.42 (0.26)	–0.02	.99
CDR-SOB^e^, mean (SD)	0.92 (1.00)	0.98 (0.85)	0.83 (1.19)	–1.24	.21
Global Deterioration Scale, mean (SD)	2.20 (0.78)	2.26 (0.75)	2.11 (0.83)	–0.78	.44
IADL^f^, mean (SD)	0.13 (0.24)	0.14 (0.21)	0.11 (0.28)	–1.09	.27

^a^VR: virtual reality.

^b^Mann-Whitney *U* tests were used for all group comparisons except for sex, which was compared using the Pearson χ^2^ test.

^c^MMSE: Mini-Mental State Examination.

^d^CDR: Clinical Dementia Rating.

^e^CDR-SOB: CDR-Sum of Boxes.

^f^IADL: instrumental activities of daily living.

### Effect of VR Cognitive Training on Visuospatial Function

Table 2 shows the comparisons of the pretraining and posttraining visuospatial function within groups, as well as group interactions in the effects of VR cognitive training. VR training resulted in significant improvement in the RCFT copy task compared to the control group. Basic components of the RCFT copy task also improved in the VR group compared to the control group.

**Table 2:** Group comparisons of visuospatial function pre and post virtual reality (VR) cognitive training.

Function score			Pretraining		Posttraining		Within groups pretraining vs posttraining				Between groups interaction^a^				
							*t (df)*		*P* value		*F*_1,39_		*P* value		*η* ^2^
**RCFT^b^copy (*z*-score^c^), mean (SD) ** ****											14.69		.001		0.30
	VR (n=23)	–0.31 (1.09)		0.22 (0.78)		–3.50 (22)		.002							
	Control (n=18)	–0.07 (1.14)		–0.47 (1.22)		2.15 (17)		.046							
**RCFT copy basic components^d^, mean (SD) **											9.27		.005		0.22
	VR	1.99 (0.59)		2.14 (0.42)		–2.82 (22)		.01							
	Control	2.15 (0.54)		2.07 (0.59)		1.53 (17)		.14							

^a^Repeated measures analysis of variance after adjusting for age (for basic components only), years of education (for basic components only), sex, Clinical Dementia Rating-Sum of Boxes, depressive symptoms, and pharmacotherapy.

^b^RCFT: Rey-Osterrieth Complex Figure Test; basic components consist of a large rectangle, diagonal cross, horizontal midline of a large rectangle, and vertical midline of a large rectangle.

^c^Adjusted for age and years of education.

^d^Raw scores.

### Effect of VR Cognitive Training on Comprehensive Neuropsychological Function

Table 3 shows the comparisons of pretraining and posttraining comprehensive neuropsychological function within groups, as well as group interactions in the effects of VR cognitive training. K-BNT, SVLT delayed recall, and Controlled Oral Word Association Test phonemic fluency showed improvement in the VR group, but the group interaction was not significant.

**Table 3:** Group comparisons of comprehensive neuropsychological tests pre and post virtual reality (VR) cognitive training.

Test^a^			Pretraining, mean (SD)	Posttraining, mean (SD)	Within groups pretraining vs posttraining		Between groups interaction^b^					
					*t (df)*	*P* value	*F*_1,39_		*P* value		*η* ^2^	
**Global cognition**												
	**MMSE^c^**							0.75		.39		0.02
		VR (n=23)	26.22 (2.91)	25.87 (3.36)	0.97 (22)	.34						
		Control (n=18)	26.28 (2.87)	26.67 (3.09)	–0.89 (17)	.39						
**Attention**												
	**Digit span, forward**							0.00		.96		0.00
		VR (n=23)	–0.11 (1.21)	–0.24 (0.87)	0.57 (22)	.57						
		Control (n=18)	–0.08 (1.08)	0.15 (1.03)	–1.42 (17)	.18						
	**Digit span, backward**						0.04		.84		0.00	
		VR (n=23)	–0.09 (0.99)	–0.15 (0.92)	0.23 (22)	.82						
		Control (n=18)	–0.23 (1.26)	–0.25 (0.82)	0.08 (17)	.94						
	**TMT^d^** **-A**							2.32		.14		0.06
		VR (n=23)	0.13 (0.58)	0.12 (0.64)	0.10 (22)	.93						
		Control (n=18)	–0.87 (4.19)	–0.38 (3.53)	–1.00 (17)	.33						
**Language and related functions**												
	**K-BNT^e^**							3.55		.07		0.09
		VR (n=23)	–0.23 (1.08)	0.19 (1.02)	–4.08 (22)	<.001						
		Control (n=18)	–0.15 (1.00)	–0.01 (1.37)	–0.72 (17)	.48						
**Verbal memory**												
	**SVLT^f^** **, immediate recall**							1.83		.19		0.05
		VR (n=23)	0.23 (0.10)	0.67 (1.24)	–3.10 (22)	.005						
		Control (n=18)	0.30 (0.83)	0.52 (0.89)	–2.29 (17)	.04						
	**SVLT, delayed recall**							3.03		.09		0.08
		VR (n=23)	–0.10 (1.40)	0.66 (1.37)	–4.59 (22)	<.001						
		Control (n=18)	0.12 (0.97)	0.58 (0.94)	–3.21 (17)	.005						
	**SVLT, recognition**							0.37		.55		0.01
		VR (n=23)	0.29 (1.39)	0.48 (1.30)	–0.93 (22)	.36						
		Control (n=18)	0.37 (1.01)	0.29 (1.07)	0.41 (17)	.69						
**Frontal executive function**												
	**COWAT^g^** **, semantic fluency**							0.04		.85		0.00
		VR (n=23)	–0.25 (0.99)	–0.44 (1.17)	1.01 (22)	.32						
		Control (n=18)	–0.41 (1.00)	–0.58 (0.88)	1.09 (17)	.29						
	**COWAT, phonemic fluency**							3.08		.09		0.08
		VR (n=23)	–0.35 (0.88)	–0.41 (0.78)	0.39 (22)	.70						
		Control (n=18)	–0.09 (0.82)	0.27 (1.01)	-1.89 (17)	.08						
	**Stroop test, color/word reading**							0.05		.82		0.00
		VR (n=23)	–0.01 (1.12)	0.32 (1.04)	–1.99 (22)	.06						
		Control (n=18)	–0.01 (0.85)	0.16 (1.21)	–0.64 (17)	.53						
	**TMT-B, mean (SD)**							0.13		.73		0.00
		VR (n=23)	–1.43 (2.04)	–0.64 (1.74)	–2.30 (22)	.03						
		Control (n=18)	–0.55 (1.52)	–0.55 (1.62)	0.01 (17)	.996						

^a^All data except for MMSE are presented as age and years of education–adjusted *z*-scores.

^b^Repeated-measures analysis of variance after adjusting for age (for MMSE only), years of education (for MMSE only), sex, Clinical Dementia Rating-Sum of Boxes, depressive symptoms, and pharmacotherapy.

^c^MMSE: Mini-Mental State Examination.

^d^TMT-B: Trail Making Test.

^e^K-BNT: Korean version of the Boston Naming Test.

^f^SVLT: Seoul Verbal Learning Test.

^g^COWAT: Controlled Oral Word Association Test.

### Effect of VR Cognitive Training on Psychiatric Symptoms

Table 4 shows the comparisons between the pretraining and posttraining measures based on psychiatric symptoms within groups, as well as group differences in the effects of VR cognitive training. Group differences were found in the AES, PANAS-P, PANAS-N, and QoL-AD measures, showing improvements in apathy, positive and negative affect, and QoL in the VR group.

**Table 4:** Group comparisons of psychiatric symptoms pre and post virtual reality (VR) cognitive training.

Group		Pretraining, mean (SD)	Posttraining, mean (SD)	Within groups pretraining vs posttraining			Between groups interaction^a^		
				*t (df)*	*P* value	*F*_1,39_		*P* value	*η* ^2^
**GDS^b^**						0.88		.36	0.03
	VR (n=23)	15.00 (6.08)	13.26 (6.49)	2.46 (22)	.02				
	Control (n=18)	12.17 (6.85)	11.72 (7.18)	0.47 (17)	.65				
**AES^c^**						7.02		.01	0.17
	VR (n=23)	47.43 (10.20)	54.35 (9.41)	–3.04 (22)	.006				
	Control (n=18)	52.83 (9.38)	51.22 (8.72)	0.98 (17)	.34				
**PANAS-P^d^**						14.40		.001	0.30
	VR (n=23)	17.00 (6.28)	21.43 (7.27)	–2.71 (22)	.01				
	Control (n=18)	21.83 (7.48)	16.50 (6.51)	4.63 (17)	<.001				
**PANAS-N^e^**						4.23		.047	0.11
	VR (n=23)	18.22 (7.09)	16.30 (6.35)	0.97 (22)	.34				
	Control (n=18)	18.89 (5.31)	20.44 (8.42)	–1.16 (17)	.26				
**QoL-AD^f^**						4.49		.04	0.12
	VR (n=18)	31.04 (4.69)	32.26 (4.96)	–1.23 (22)	.23				
	Control (n=23)	34.94 (9.43)	32.72 (6.54)	1.21 (17)	.25				

^a^Repeated-measures analysis of variance after adjusting for age, years of education, sex, Clinical Dementia Rating-Sum of Boxes, and pharmacotherapy.

^b^GDS: Geriatric Depression Scale.

^c^AES: Apathy Evaluation Scale.

^d^PANAS-P: Positive and Negative Affect Schedule-positive affect.

^e^PANAS-N: Positive and Negative Affect Schedule-negative affect.

^f^QoL-AD: Quality of Life-Alzheimer Disease.

### Simulator Sickness, Interest, and Satisfaction Associated with the VR Training Program

Table 5 shows the simulator sickness measured by the SSQ after each training session, reported on a Likert scale ranging from 0 to 100, in the VR group participants after the training period. The mean SSQ total score was 12.86 (SD 11.82), and the summary subscale mean score for nausea, oculomotor, and disorientation was 7.02 (SD 6.40), 11.15 (10.56), and 17.16 (16.91), respectively.

 Interest and satisfaction had mean scores of 79.78 (SD 14.18) and 78.04 (SD 12.50) on a Likert scale ranging from 0 to 100, respectively.

**Table 5:** Mean (SD) simulator sickness questionnaire scores associated with the virtual reality cognitive training (n=23).

Session	Nausea	Oculomotor	Disorientation	Total score
1	9.95 (14.80)	14.83 (14.18)	22.39 (22.89)	17.24 (17.53)
2	6.22 (10.61)	11.53 (16.30)	15.13 (18.25)	12.20 (15.59)
3	9.13 (12.69)	11.21 (16.62)	16.95 (25.51)	13.66 (18.86)
4	6.64 (9.73)	9.89 (14.89)	10.29 (19.78)	10.24 (14.97)
5	6.64 (10.54)	7.91 (13.02)	13.92 (23.37)	10.24 (14.63)
6	2.45 (7.17)	9.56 (15.70)	20.58 (26.83)	11.22 (16.18)
7	5.39 (6.94)	9.89 (12.61)	19.37 (27.12)	12.20 (14.93)
8	9.97 (11.20)	14.82 (17.27)	18.98 (27.35)	16.32 (19.47)

### Increased Functional Connectivity in rsfMRI

We investigated brain functional connectivity in the visual network associated with the improvement in the RCFT copy task. The areas with significantly increased connectivity in the seed-to-voxel visual networks are presented in Table 6: (a) from the right visual lateral cortices to the left paracingulate gyrus, right paracingulate gyrus, left frontal pole, left superior frontal gyrus, anterior cingulate gyrus, and white matter; and (b) from the visual medial cortices to the right insular cortex, right frontal pole, right frontal operculum cortex, right caudate, left caudate, right putamen, left insular cortex, and white matter.

**Figure 2:**
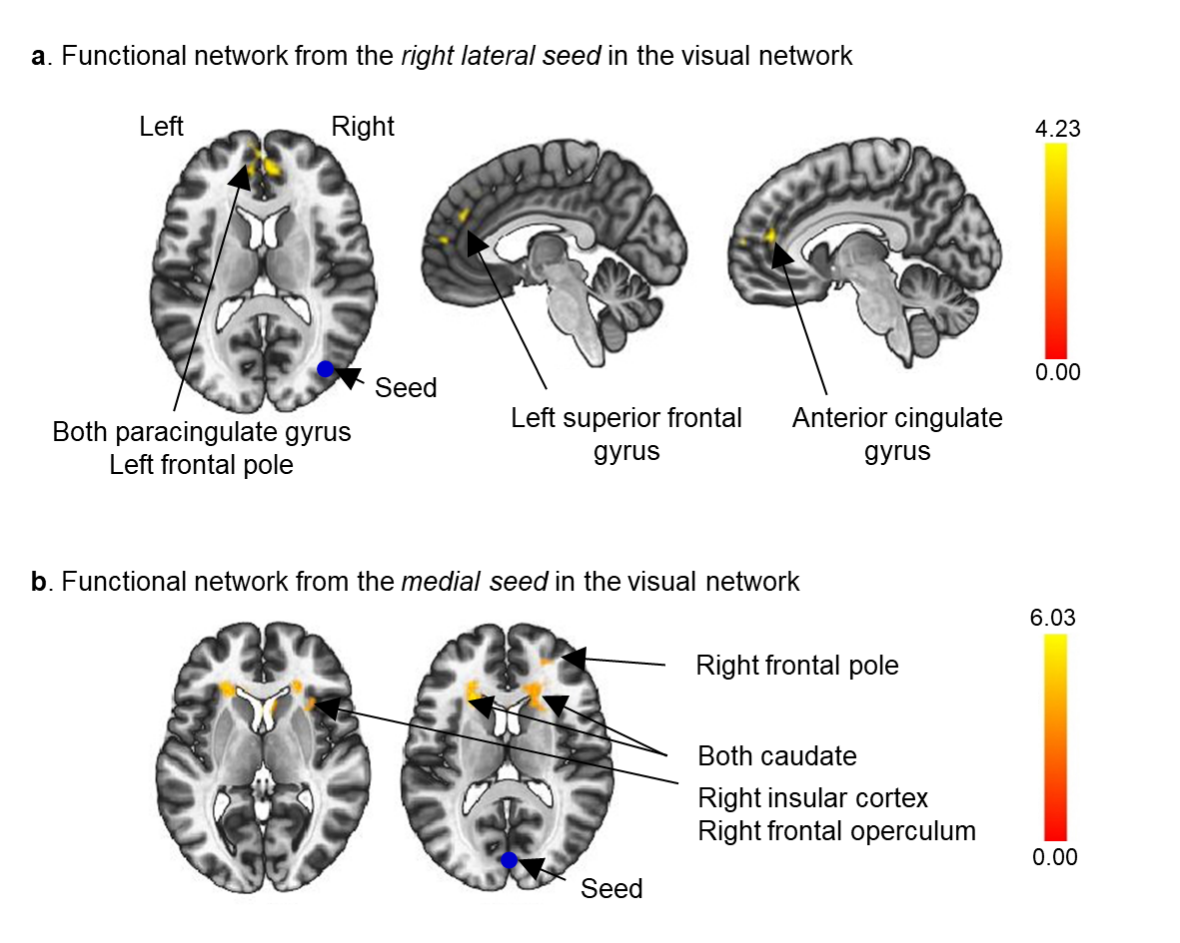
Seed-to-voxel analyses based on the right lateral region (a) and the medial region (b) of the visual network (blue circles). Increased frontal-occipital functional connectivity related to the Rey-Osterrieth Complex Figure Test copy task improvement after virtual reality cognitive training. False discovery rate–corrected *P*<.05 for cluster threshold; uncorrected *P*<.001 for voxel threshold.

Figure 2 depicts the increased regional functional connectivity in the brain cortices and the white matter that are related to improvements in the RCFT copy task in the VR group compared to the control group.

**Table 6:** Functional visual network connectivity related to improved Rey-Osterrieth Complex Figure Test copy task scores after virtual reality cognitive training.

Seed and connected regions (voxels)		Clusters		
		Voxel (2×2×2)	MNI^a^ coordinates (x, y, z)^b^	FDR^c^–corrected *P* value^d^
**Visual lateral, R^e^**		291	–06, +40, +42	.003
	Paracingulate gyrus, L^f^	118		
	Paracingulate gyrus, R	68		
	Frontal pole, L	41		
	Superior frontal gyrus, L	29		
	Anterior cingulate gyrus	7		
	Frontal pole, R	1		
	White matter	27		
**Visual medial**		719 and 401	+16, +20, +16 and –22, +22, +16	<.001 and <.001
	Insular cortex, R	71		
	Frontal pole, R	48		
	Frontal operculum cortex, R	25		
	Caudate, R	24		
	Caudate, L	3		
	Putamen, R	2		
	White matter	546		
	Insular cortex, L	2		
	White matter	399		
Visual lateral, L		N/A^g^	N/A	N/A
Visual occipital		N/A	N/A	N/A

^a^MNI: Montreal Neurological Institute.

^b^Coordinates indicate the representative coverage region with maximum power among all connected regions.

^c^False-discovery Rate.

^d^Group-level analyses between the VR and control groups were performed using a general linear model with Rey-Osterrieth Complex Figure Test copy task improvement as an explanatory variable and the post-pre training *z* transformation value as a dependent variable after adjusting for age, years of education, sex, Clinical Dementia Rating-Sum of Boxes, depressive symptoms, and pharmacotherapy.

^e^R: right side.

^f^L: left side.

^g^N/A: not applicable.

## Discussion

### Principal Findings

This study found that 1-month multidomain cognitive training using fully immersive VR was effective in improving visuospatial function and frontal-occipital functional connectivity, as well as apathy, affect, and QoL in older people in a predementia cognitive state.

The first major finding of this study is that VR cognitive training resulted in improvements in the RCFT copy task. Despite the inconsistent results reported in the literature, training-related changes in cognition have been repeatedly found in older people with cognitive disorders [9,10]. Neuropsychological test score improvements after traditional pen-and-paper or computerized cognitive training have been found in measures of global composite cognition [46-48], verbal memory [11,46,49,50], verbal letter fluency [46,47], verbal fluency [51,52], and visuospatial function in the clock-drawing test [46,53]. It has also been reported that VR cognitive training was effective in improving frontal executive function in individuals with MCI [54], as well as attention and visual memory in older people [55,56]. In line with these previous studies, our results also showed that multidomain cognitive training in a virtual environment was effective in visuospatial function measured by the RCFT copy task in total and basic components comprising the gestalt of the feature showing the ability to approach [26,27]. Although not significant, improvements in naming ability, verbal memory delayed recall, and frontal executive function were also found in the training group compared to the control group. It is possible that the relatively short 1-month training period might have resulted in the lack of group difference, as a learning effect may have impacted the posttraining neuropsychological test results in the control group. However, the improvement in visuospatial function in the VR group, even after the short period of cognitive training, might be attributed to the ecological nature of the fully immersive VR environment. In the enriched auditorily and visually stimulating environment, processing of visual orientation, visuospatial construction, and visual selective attention likely occurred [57,58]. In recent studies with VR evaluation, investigators have been able to effectively differentiate between the navigational [59] and visuospatial deficits observed in patients with MCI and healthy older people [60,61]. In studies with VR interventions, VR cognitive training was found to be effective [55,62] or ineffective [56,63] in improving visuospatial function in older people or those in an early dementia stage. We believe that the cognitive training performed in the maximally immersive environment with the head-mounted display, headphones, and hand movement trackers in our study might have increased visuospatial functioning in those at a predementia stage [64]. The immersion methods utilized in previous studies investigating VR cognitive training in older people have employed desktop-based systems [55,56], screen and sensors [62], screen and glasses [65,66], and head-mounted display and fixed joystick setups [63]. Although heterogeneity in study populations and methodological differences among prior studies have resulted in inconsistent findings, this study provides further evidence to support the benefits of VR cognitive training in eliciting improvements in visuospatial processing through the repeated presentation of real-world, dynamic, multisensory, and interactive environments.

Another novel finding was the increased functional connectivity observed in the frontal-occipital cortical network after VR cognitive training, which was associated with improved performance in the RCFT copy task, consistent with the associations between cognitive improvements and neuronal plasticity that have been observed previously [67]. In patients with MCI, significant associations have been observed between verbal memory improvement and left hippocampal activation in task-related fMRI after 8-week training to improve auditory processing speed and accuracy [11]. Other studies have shown that 6 weeks of episodic memory training in patients with MCI resulted in the manifestation of new associations between improved delayed word recall test performance and brain activation in the right inferior parietal lobule in fMRI during memory encoding [68]. In healthy older people, 8 weeks of exposure to a cognitive control training program led to an increased frontoparietal network related to cognitive control ability [69]; another study found that verbal recall was associated with an increased left hippocampal volume in healthy older people after 8 weekly verbal recall memory training sessions [70]. Thus, in this study, repetitive cognitive training in a novel fully immersive environment might have increased the frontal-occipital activation in accordance with improved visuospatial function. We also observed increased functional connectivity in white matter areas, which are known to exhibit a lower hemodynamic response than the grey matter. Although fMRI studies have focused on grey matter until recently, the increased functional connectivity in the white matter close to the grey matter supports the growing neural evidence of fMRI white matter changes induced by VR cognitive training [71,72].

This evident link between visuospatial construction and frontal-occipital functional connectivity might be explained by the acquired cognitive system engagement induced by the RCFT copy task, which requires the participant to copy a complex geometric figure [73]. Visuoconstructive ability is based on the Van Sommers model of drawing [74]; according to this cognitive model, the RCFT copy task consists of (i) visual recognition of a 2D Rey-Osterrieth complex figure; (ii) visual representation of the figure in long-term or temporary memory; (iii) graphical output processes such as those related to depiction decisions (eg, context, orientation, viewpoint, details, and boundary) or reproduction strategies (eg, copying orders, dimensions, shapes, diagonals, crosses, line sets); (iv) graphical planning (eg, routine or contingent planning); and (v) articulation and economic constraints during motor output. Through these steps, multiple brain regions have been found to be associated with performance in the RCFT copy task, including the temporal, parietal, occipital, and frontal cortices in both hemispheres or in the right hemisphere alone [75-77]. Although we observed increased activity only in the primary visual cortices (visual medial) and the right associative visual cortices (right visual lateral) connecting to the areas in the middle frontal cortices, these regions are known to be involved in the visual recognition and graphic output planning processes required to complete the RCFT copy task [74], and are associated with visuo-motor transformation and multistep object use in the task [77]. A recent study reported that lesions in the right superior parietal lobe and the middle occipital gyrus were associated with poor RCFT copy task performance [78], which is in accordance with our results. Furthermore, there have been reports on improvements in nontrained cognitive functions, also known as transfer effects, in memory training in older people with MCI [79,80]. Previous studies have shown that repeated memory-focused training might have enhanced the processing speed of memory retrieval and the efficiency of working memory, assuming that frontal executive function was the main recipient of the transfer effects [79,80]. Although recent studies have applied cognitive training with novel computerized tools and involvement of multiple cognitive domains, existing programs have only applied cognitive training in a 2D environment with an emphasis on language abilities [9,53,79,81]. Since frontal executive function plays a major role in all cognitive domains and higher-order cognitive controls [82], the improved performance on the RCFT copy task may be supported by increased functional connectivity in the frontal-occipital network.

The psychiatric benefit of VR cognitive training in individuals in a predementia state should be considered. In this study, participants in the VR group showed improved apathy, affect, and QoL scores after training compared with those in the control group. A recent review reported that computerized cognitive training resulted in long-term improvements in psychological outcome measures [16]. Although methodologies vary across studies, 3D VR cognitive training was effective in improving depressive symptoms in patients with MCI compared with an active control group receiving music therapy [63]. Moreover, a few feasibility studies have reported improved alertness, pleasure, apathy, and security following one-time exposure to a less immersive VR environment [65,66]. We postulate that apathy, affect, and QoL might be improved by the VR cognitive training, as these are some of the early symptoms of dementia [83]. Immersive virtual environments might facilitate the limited functioning of patients with cognitive disorders that affect communication, interaction, motivation, engagement, and positive attitudes toward others [84]. Thus, the importance of virtual environments should be considered in cognitive training because the feeling of presence itself in a 3D space can enhance volitional motivation, allowing one to constantly process external stimuli and cognitively adjust to changing environments [85].

Simulator sickness reported after every session was minimal in the VR cognitive training group. In this study, the SSQ total score (mean 12.86, SD 11.82) indicated minimal symptoms (score 5-10) according to the suggested categorization established in flight simulators [86]. Although the SSQ was originally developed and validated in military personnel using flight simulators, it is the most commonly used measure of sickness in a virtual environment [40]. A recent meta-analysis of the SSQ in virtual environments reported total and subscale scores and dropout rates according to VR conditions (total 28.00, SD 1.71; nausea 16.72, SD 0.77; oculomotor 17.09, SD 0.55; disorientation 23.50, SD 1.17; dropout rate 15.6%) [87]. Compared to the results in a VR environment, our results on the SSQ scores and dropout rates (8%) showed better tolerability, with moderate interest and satisfaction. The SSQ scores have been reported to be higher in VR than in a flight simulator environment [86] and with gaming content than without it [87]. Despite the characteristics of VR in this study, such as fully immerse, game and training components, and old users at an early stage of cognitive decline, these results may imply that fully immersive VR can be a safe and interesting method for cognitive training.

### Limitations

Our study had several strengths and limitations. This is one of the largest VR cognitive training studies to use a fully immersive 3D VR program. Compared to 2D or semi-immersive VR programs, our results highlight the positive effects of employing fully immersive 3D VR in cognitive training, as we found neural evidence supporting the improvement in visuospatial function. However, there are several limitations and lessons learned in this study. First, the small sample size and short training period were the main limitations. Although sample sizes in studies investigating the effects of cognitive training are increasing [88], most VR trials still rely on small sample sizes and are performed over a short duration, especially those using fully virtual environments [15]. Short clinical trial periods in previous studies investigating the effect of computerized cognitive training programs have also been a limiting factor in the field as a whole [88]. Thus, future studies should aim to increase the sample sizes and extend the duration of training to better evaluate the effect of VR cognitive training. Second, we considered that the per-protocol analysis could bias the results of this randomized controlled trial, although the number of participants who dropped out of the study was the same in both groups. Third, the lack of an active control group in this study is another limitation. Some previous trials have included active control groups receiving psychoeducation, cognitive therapy, face-to-face music therapy, or pen-and-paper cognitive training for comparisons with the VR training group [15,63]. In the future, various active control groups should be considered to confirm the effectiveness of VR cognitive training. Fourth, the lack of examination for AD biomarkers such as cerebrospinal fluid analysis or brain imaging for amyloid detection can be a limitation because it is unclear whether the participants in our study will develop AD, which is the most prevalent cause of dementia. Future studies involving AD biomarkers could clearly explain the pure effect of cognitive training in individuals in a preclinical or prodromal dementia state. Lastly, heterogeneity among patients, practitioners, program content, and accessibility to the VR system can limit the generalizability of the results to other populations.

### Conclusions

We found that fully immersive VR cognitive training improved cognition and psychiatric symptoms in a predementia state. Visuospatial function improved in such individuals relative to controls, and this finding was supported by increased frontal-occipital functional connectivity assessed by rsfMRI. These findings suggest that VR training can enhance visuospatial ability by exposing patients to an enriched virtual environment, leading to improved apathy, affect, and QoL. Our results support the neurotherapeutic use of VR cognitive training as an effective nonpharmacological intervention for those who are at risk for dementia; however, more rigorous trials should be performed to confirm the effects and identify the associated neural mechanisms.

## Appendix

Multimedia Appendix 1 CONSORT-EHEALTH (V 1.6.1) - Submission/Publication Form.

Multimedia Appendix 2 Representative images of the virtual reality training program.

## Abbreviations

AD: Alzheimer disease
AES: Apathy Evaluation Scale
CDR: Clinical Dementia Rating
CDR-SOB: Clinical Dementia Rating-Sum of Boxes
fMRI: functional magnetic resonance imaging
GDS: Geriatric Depression Scale
ICT: information and communication technology
K-BNT: Korean version of the Boston Naming Test
MCI: mild cognitive impairment
MMSE: Mini-Mental State Examination
MRI: magnetic resonance imaging
PANAS: Positive and Negative Affect Schedule
PANAS-N: Positive and Negative Affect Schedule-negative
PANAS-P: Positive and Negative Affect Schedule-positive
QoL: quality of life
RCFT: Rey-Osterrieth Complex Figure Test
rsfMRI: resting-state functional magnetic resonance imaging
SSQ: Simulator Sickness Questionnaire
SVLT: Seoul Verbal Learning Test
TMT: Trail Making Test
VR: virtual reality
