# Supplementary material for: Effect of Cognitive Training in Fully Immersive Virtual Reality on Visuospatial Function and Frontal-Occipital Functional Connectivity in Predementia: Randomized Controlled Trial
Source: J Med Internet Res. 2021 May 6;23(5):e24526. doi: 10.2196/24526 (PMC8138710; doi:10.2196/24526)
Supplement: Multimedia Appendix 2 [file jmir_v23i5e24526_app2.docx]

**
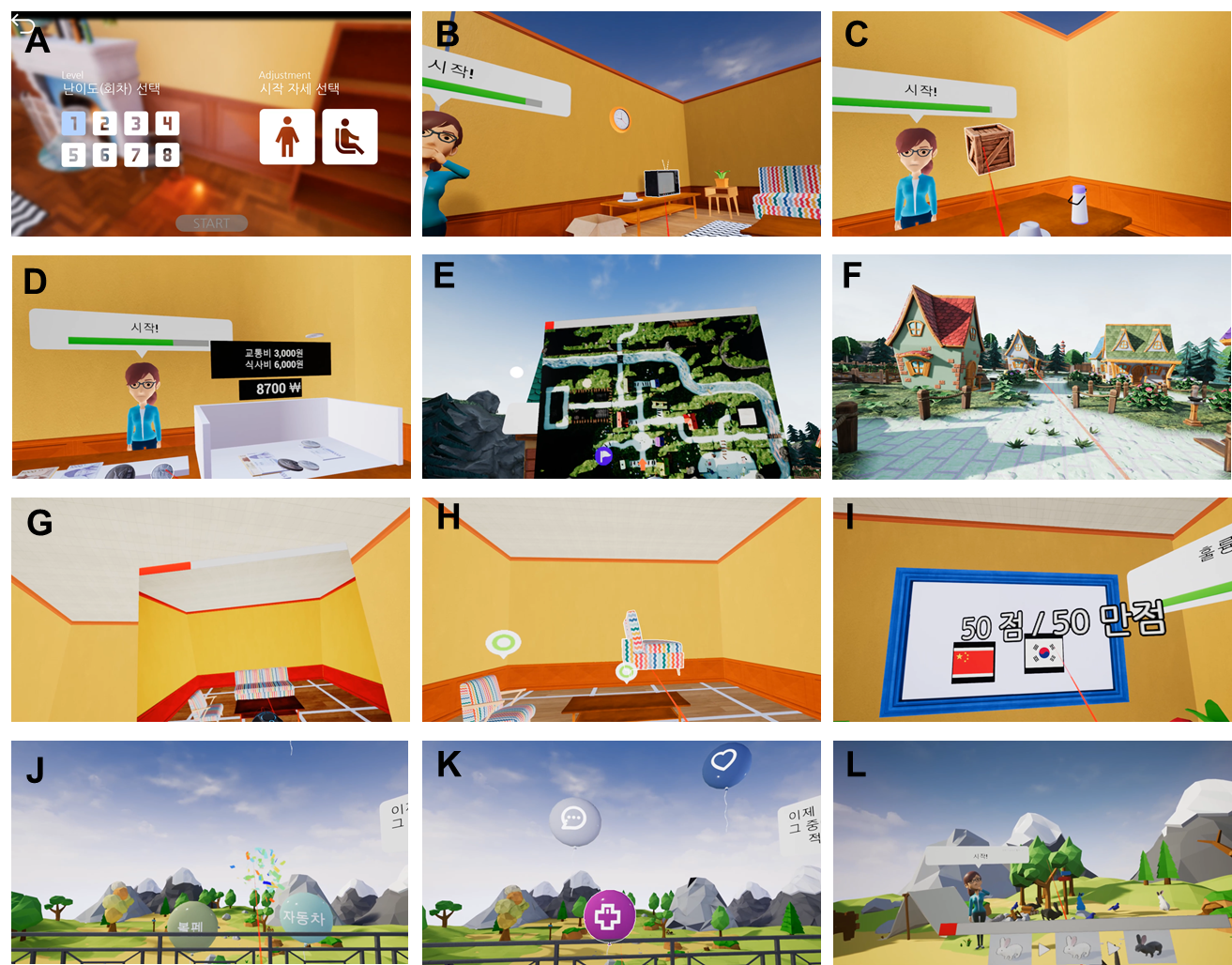
**

(A) Starting options with 8 levels and 2 positions; (B) to find differences; (C) to pick items needed for certain tasks; (D) to prepare an exact amount of money; (E) 10 seconds of memorizing a map; (F) path finding without a map; (G) 10 seconds of memorizing a picture of a room; (H) to place furniture exactly like the picture; (I) to remember the flags; (J) to remember and recall the words; (K) to remember and recall the symbols; (L) to catch animals in a certain order.
